# Heterotopic Gastric Mucosa Causing Rectal Bleeding in a Young Child

**DOI:** 10.1097/PG9.0000000000000184

**Published:** 2022-02-25

**Authors:** Blake Patricia-Rae Meyer, Johnny Nguyen, Michael Wilsey, Sara Karjoo

**Affiliations:** * Florida State University College of Medicine, Tallahassee, FL; † Department of Pathology, Johns Hopkins All Children’s Hospital, St. Petersburg, FL; ‡ Department of Gastroenterology, Johns Hopkins All Children’s Hospital, St. Petersburg, FL.

**Keywords:** heterotopic gastric mucosa, hematochezia, eosinophilic esophagitis

## Abstract

Heterotopic gastric mucosa (HGM) in the colon and small bowel is a very rare finding. We report a case of HGM in the rectum of an 8-year-old child with a history of eosinophilic esophagitis after having a colonoscopy to evaluate for inflammatory bowel disease. The colonoscopy was normal except for rectal tissue erythema and edema. Inflammatory bowel disease has been reported in some cases of children with eosinophilic changes of the esophagus. The child had intermittent rectal bleeding thought to be due to constipation. Interestingly, when the patient was placed on a proton pump inhibitor for the treatment of eosinophilic esophagitis, the rectal bleeding decreased. After our patient ceased proton pump inhibitor therapy, he experienced a large amount of rectal bleeding. Histological findings revealed HGM in the colon/rectum. An extensive review of the incidence, diagnosis, and treatment is discussed.

## INTRODUCTION

Heterotopic gastric mucosa (HGM) located in the rectum is very uncommon.^1^ It is more likely to be found in the esophagus, small intestine, or with a Meckel’s diverticulum.^1^ When in the rectum, HGM is typically found 5 to 8 cm from the anal verge.^1^ A systematic review in 2017 by Iacopini et al found 78 cases, including the first pediatric case described in 1939, with a total of 34 pediatric cases.^2^ The median age at diagnosis was 22 years, and 63% of cases were males.^2^ The prevalence and incidence of HGM overall in children is limited in the literature, but it has been noted in various locations throughout the gastrointestinal tract, and presentations range from asymptomatic to recurrent epigastric pain, recurrent pancreatitis, or chronic gastrointestinal bleeding depending on the location of the HGM.^3^ One retrospective study identified the endoscopic prevalence of HGM in the proximal esophagus to be 1.4% out of 1399 pediatric patients, while a systemic review identified 31.4% of 34 cases of HGM in the gallbladder to be in children.^4, 5^ HGM is present in nearly half of Meckel’s diverticulum cases with up to 80% being symptomatic, most commonly with painless rectal bleeding.^6^ The pathogenesis of HGM is still unclear, but it is thought to be derived either congenitally during organogenesis (heteroplasia) or acquired from the repair of injured epithelium (metaplasia).^1^

HGM often occurs as a solitary lesion in the rectum but can be multifocal.^2^ The most commonly reported symptom of HGM in the rectum is hematochezia, but other symptoms include anal pain, tenesmus, burning, pruritis ani, bowel habit changes, bloating, discomfort in the lower abdomen, and cramping.^2^ Pediatric patients are more likely to report specific anorectal symptoms and are more likely to have HGM-related complications, such as ulcers, fistulas, or bowel perforation.^2^ These symptoms may mimic inflammatory bowel disease (IBD). Histopathological examination most commonly demonstrates oxyntic-type gastric mucosa.^2^

We report a case of unusual histological finding of HGM in the rectum in an 8-year-old child after colonoscopy to evaluate for IBD.

## CASE PRESENTATION

The patient is an 8-year-old male with a history of constipation, eosinophilic esophagitis (EoE), and failure to thrive, who presented with worsening intermittent hematochezia suspected to be due to constipation. He was initially treated with ranitidine, omeprazole, polyethylene glycol, and a high-fiber diet, with resolution of hematochezia. Regurgitation and reflux continued despite treatment, and EoE was diagnosed at 6 years via upper endoscopy. The patient’s family elected to stop the proton pump inhibitor (PPI) and initiated diet elimination intervention for EoE, which excluded gluten, dairy, soy, egg, nuts, and fish/shellfish. Oral swallowed steroids were not prescribed, as family wanted treatment without medicines. His cough and regurgitation improved on diet, but hematochezia recurred with mucous-filled stools. Differential diagnosis at this time included constipation, fissures, infectious colitis, Meckel’s diverticulum, vascular ectasias, bleeding disorder, rectal ulcers, polyps, and IBD. Stool samples were negative for microorganisms, and calprotectin was normal. Polyethylene glycol was used to rule out constipation and fissures. However, the rectal bleeding worsened with blood dripping down his leg after bowel movements.

**FIGURE 1.**
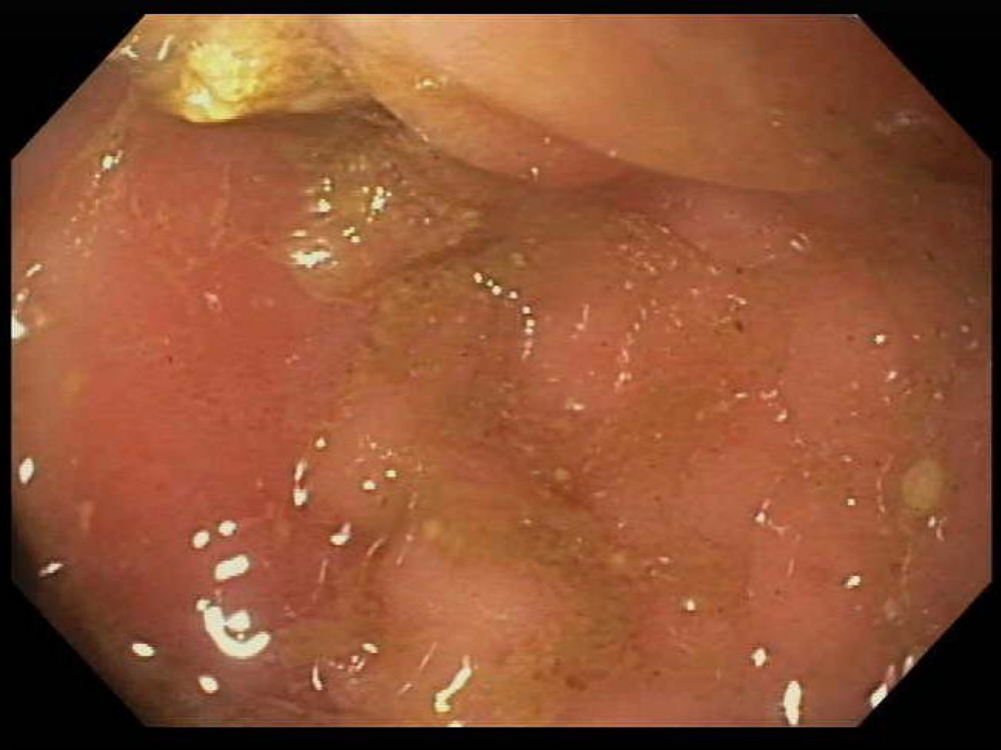
Endoscopic view of rectum showing edema, erythema, and loss of vascularity. No ulcerations were seen.

**FIGURE 2.**
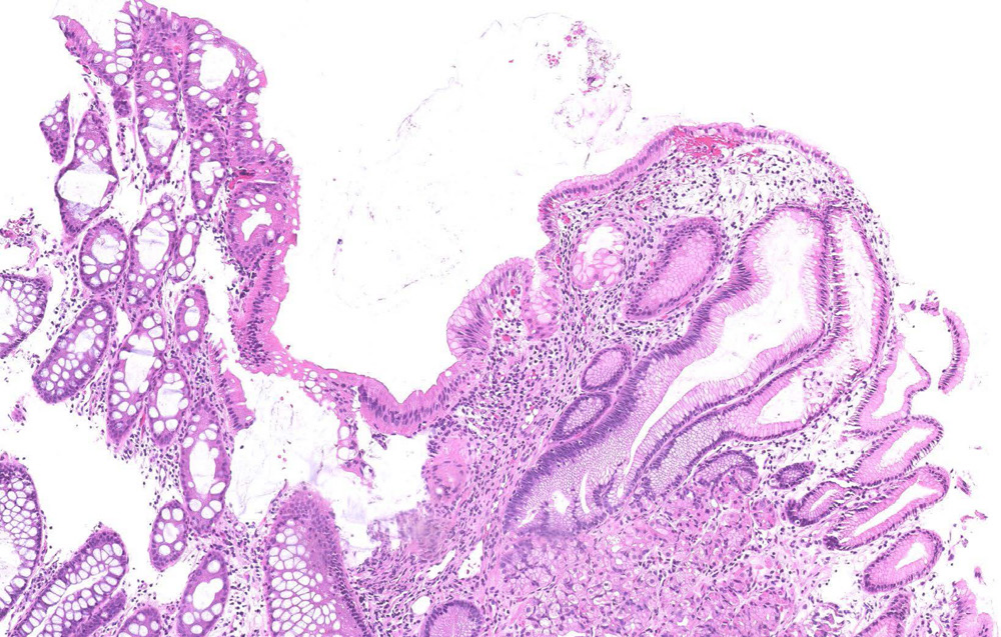
Hematoxylin and eosin–stained histologic section (×100 total magnification) revealing abrupt transition between normal colorectal mucosa (left side of the field) to abnormal oxyntic (fundic) type gastric heterotopic mucosa (right side of the field). No inflammation, granulomas, or microorganisms detected.

**FIGURE 3.**
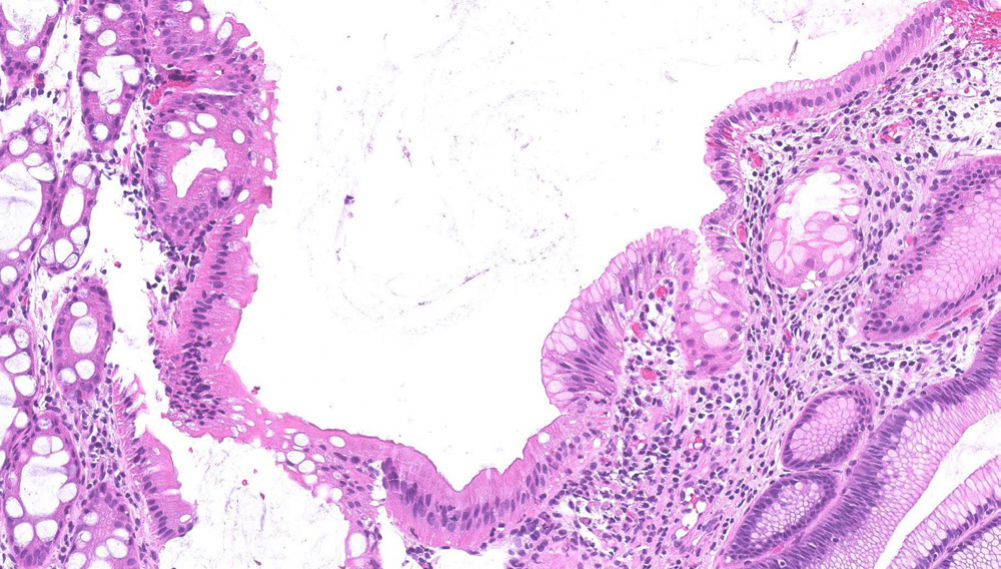
Hematoxylin and eosin–stained histologic section (×200 total magnification), higher-power view showing surface epithelial transition between normal colorectal enterocytes (left side of the field) to gastric foveolar epithelium (right side of the field).

Due to worsening hematochezia and mucous-filled stools, repeat esophagogastroduodenoscopy and colonoscopy were performed to evaluate for the etiology of rectal bleeding. The mucosa of the terminal ileum and entire colon was grossly normal, except for edema and erythema of the rectum (5 cm from the anal verge) without ulceration or nodularity (Fig. 1). Histology of the rectum demonstrated heterotopic gastric oxyntic (fundic) mucosa in continuation with colonic and rectal mucosa (Figs. 2, 3). There was no evidence of granulomas, cryptitis, microorganisms, dysplasia, or malignancy. There were no eosinophils in the mucosa. The sample of HGM tested negative for *Helicobacter pylori* by Warthin Starry special stain and immunohistochemical staining.

Following the discovery of the HGM in the rectum, a Meckel scan was performed with technetium-99m (and priming with the H2 blocker before scan), which did not show abnormal radiopharmaceutical uptake in the descending colon, rectum, or other parts of the gastrointestinal tract. The patient was restarted on PPI, and the family is currently considering their options for treatment of the HGM.

## DISCUSSION

Rectal bleeding can be from various causes including anal fissures secondary to constipation, infections, IBD, polyps, and rarely Meckel diverticulum, vascular anomalies, bleeding disorders, and rapid upper gastrointestinal bleeding. However, recent research has shown possible associations between IBD and EoE^7^ and may be a source of rectal bleeding in patients with EoE. The recent retrospective case-control study found the prevalence of IBD among pediatric EoE patients to be significantly higher than EoE and IBD alone in the general population (2.2% compared to 0.4% in the general population).^7^ This association and other etiologies of rectal bleeding prompted the clinical team to proceed with colonoscopy, leading to the diagnosis of HGM in the rectum. Clinical features of HGM can mimic IBD and may be difficult to differentiate without histological evaluation. Children with HGM presented most often with grossly aberrant mucosa (45%), solitary ulcers (33%), or polyps (22%) endoscopically.^8^ In a systematic review of 78 cases, only 5 cases reported multifocal localization of HGM.^2^ Direct histologic evaluation may be the best diagnostic test. Our patient had a negative Meckel scan despite the pathology biopsy showing HGM located in the rectum. False negative scans are possible if the location of HGM is obscured by the bladder or if the size of the HGM is too small.^9^ Clinicians should recognize that a normal Meckel scan does not exclude small foci of HGM. A Meckel scan in 9 pediatric cases of HGM demonstrated only 3 positive scans with uptake in the rectum.^2^ From these studies, a Meckel scan may be low yield to identify HGM.

Previous studies have shown that PPIs or H2 receptor antagonists can be effective at temporary symptom control or ulcer healing, with many cases ultimately reporting surgical excision of the HGM.^2^ Following surgical excision for larger lesions (median size, 25 mm) or endoscopic removal (median size, 20 mm) in 60 cases, the symptoms did not return after follow-up ranging from 2 to 84 months (median, 22 months).^2^ Pediatric rectal HGM cases in the literature were often treated with transanal surgery.^8^ At least 4 pediatric cases have also been treated with endoscopic mucosal resection without complications.^10^

There is inconclusive evidence about whether HGM can progress to malignancy, but the potential is present. There are only 3 adult cases in the literature reporting rectal HGM with metaplasia.^2^ No pediatric cases of malignancy have been reported. Additionally, HGM has been shown to progress to adenocarcinoma or dysplasia in other organs, such as the esophagus or gallbladder.^11^ The cause of malignant transformation of HGM is not well understood, with some proposing that *H pylori* infection is involved and that progression is stepwise from metaplasia to dysplasia to adenocarcinoma.^12^ Our patient’s tissue sample was negative for *H pylori*. The prognosis and evolution of HGM in the rectum remains unclear, so endoscopic surveillance may be needed to continue to monitor the affected area if it is not resected. Our patient is currently considering various treatment options and will be monitored by the clinical team closely.

## ACKNOWLEDGMENTS

B.P.-R.M., J.N., and S.K. contributed a significant portion of drafting the manuscript and approved the final draft submitted. M.W. also edited the document. B.P.-R.M. obtained patient data and contributed greatly to the final manuscript. This includes writing and editing of the manuscript. J.N. contributed to pathology figures and writing of the manuscript. S.K. provided supervision, expertise on the topic, idea creation, and writing and editing of the manuscript. M.W. edited the document and provided feedback. All authors read and approved the final manuscript. S.K. is the guarantor of the article.
